# Surgical Options for Primary Synovial Chondromatosis of the Knee: A Systematic Review

**DOI:** 10.1016/j.artd.2025.101796

**Published:** 2025-08-13

**Authors:** Zhen Jonathan Liang, Yu Liu, Vachalam Rangasamie Danakkrisna, Zi Qiang Glen Liau

**Affiliations:** a Department of Orthopaedic Surgery, National University Hospital, Singapore; b Department of Orthopaedic Surgery, Alexandra Hospital, Singapore

**Keywords:** Synovial Chondromatosis, Knee, Arthroscopy, Total knee arthroplasty, Synovectomy

## Abstract

**Background:**

Synovial chondromatosis is a rare benign condition characterized by the formation of cartilaginous nodules within the synovium of joints, bursae, or tendon sheaths. While nonsurgical management exists, surgical intervention is often necessary. This systematic review aims to evaluate surgical options for managing synovial chondromatosis of the knee and compare recurrence rates following each procedure.

**Methods:**

Adhering to the Preferred Reporting Items of Systematic Review and Meta-Analysis guidelines, a literature search was conducted using PubMed, SCOPUS, and Embase, up to April 2025. Studies included detailed surgical interventions for primary synovial chondromatosis of the knee.

**Results:**

Thirty-seven studies met the inclusion criteria, comprising 30 case reports, 4 retrospective studies, and 3 case series, involving 120 patients (70 males, 49 females, 1 unspecified). Surgical techniques were categorized into open, arthroscopic, and combined approaches. Open approaches included arthrotomies without synovectomy, partial and total synovectomies, and total knee arthroplasty. Arthroscopic approaches involved washouts and synovectomies (partial and total). Combined approaches included staged surgeries with both open and arthroscopic techniques. Recurrence rates varied: open arthrotomy (0%; 1 and 20 year follow-up), arthroscopic synovectomy (5.71%; average follow-up duration 1.53 years), open synovectomy (12.5%; average follow-up 2.04 years), total knee arthroplasty (20.8% average follow-up 10.3 years), and arthroscopic washout (31.4%; average follow-up 6.56 years). Combined approaches reported no recurrences in 6 cases with average follow-up 1.44 years.

**Conclusions:**

This systematic review highlights variability in recurrence across techniques. Arthroscopic synovectomy and combined approaches demonstrated the lowest recurrence; however, the choice of surgical method should be tailored to the individual patient's condition and the surgeon's expertise.

## Introduction

Synovial chondromatosis is a rare benign condition characterized by the formation of cartilaginous nodules within the synovium of joints, bursae, or tendon sheaths [[1], [2], [3]]. The knee joint is one of the most commonly affected sites [4]. This condition can cause significant morbidity due to pain, swelling, and mechanical symptoms, often leading to functional impairment.

It is crucial to note that the clinical presentation of synovial chondromatosis can vary widely, with differences in the number, size, and location of loose bodies, as well as the duration of symptoms prior to surgery [5]. These factors may significantly impact both the selection of the surgical technique and the risk of recurrence.

Synovial chondromatosis can be defined as primary or secondary. Secondary synovial chondromatosis would arise as a result of mechanical changes in a joint due to arthropathy, while primary synovial chondromatosis formation of multiple chondroid nodules and osteochondral or osseous loose bodies without a secondary pathological process [6,7].

Management of synovial chondromatosis, particularly in the knee, involves both nonsurgical and surgical approaches. Conservative treatment may be trialled [8] but surgical intervention is often required to relieve symptoms and prevent recurrence [9] given the limitation of range of motion and recalcitrant swelling [6,10]. Various surgical techniques have been described, including open synovectomy [11], arthroscopic synovectomy [12,13], open arthrotomy [14] for removal of loose bodies and total knee arthroplasty (TKA) should there be secondary osteoarthritis [15].

Recurrence of synovial chondromatosis after initial attempts at removing loose bodies has been documented in the literature [[16], [17], [18]] and the controversy of whether a synovectomy should be included to prevent recurrence has been widely debated [[18], [19], [20], [21], [22]].

This systematic review aims to summarize the existing literature and evaluate the surgical options for the management of synovial chondromatosis of the knee and compare the rate of recurrence following each procedure.

## Material and methods

### Data collection

This study was conducted based on the Cochrane Handbook for Systematic Reviews of Interventions version 6.3 [23]. Reporting of results is in accordance with the Preferred Reporting Items of Systematic Review and Meta-Analysis statement [24]. This study has also been registered under PROSPERO (CRD42024551236).

A literature search through PubMed, SCOPUS, and Embase was conducted. Eligible studies included those from the start of available publications published up to April 2025. The search strategy aimed to identify all relevant studies on the surgical management of synovial chondromatosis of the knee arthroscopic synovectomy, open synovectomy, and TKA and can be found in Table 1.

**Table 1:** Search strategy employed.

PubMed - 243 results	(“Synovial Chondromatosis”[Title/Abstract] OR (“Synovial”[Title/Abstract] AND “Chondromas”[Title/Abstract])) AND “Knee”[Title/Abstract]
SCOPUS: 448 results	(TITLE-ABS-KEY (synovial AND chondromatosis) AND TITLE-ABS-KEY (knee))
Embase: 299 results	“synovial chondromatosis”:ti,ab,kw AND knee:ti,ab,kw

**Figure 1:**
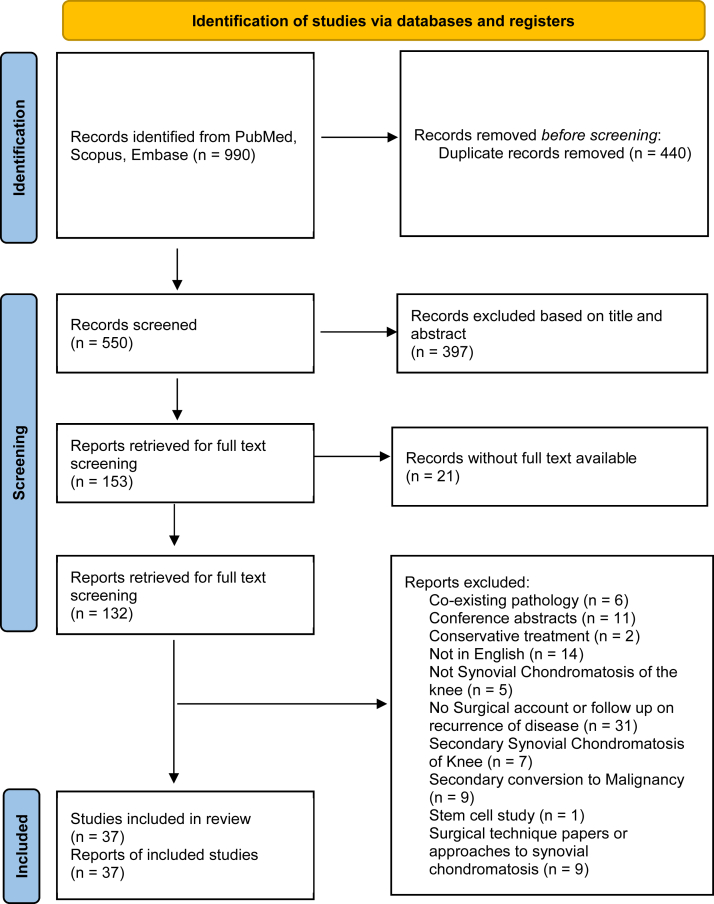
PRISMA diagram. PRISMA, Preferred Reporting Items of Systematic Review and Meta-Analysis.

The search workflow adhered to the Preferred Reporting Items for Systematic Reviews and Meta-Analyses guidelines [24] and is illustrated in Figure 1. To identify studies to be included in the final review, the articles were independently assessed by authors to determine eligibility for inclusion in the analysis. Any disagreements were resolved by consensus discussion among the authors.

A total of 990 records were obtained from database screening. After removing 440 duplicates, 550 records were screened based on titles and abstracts, resulting in the exclusion of 397 records. The full texts of 153 articles were retrieved for further evaluation. Twenty-one articles were excluded due to the unavailability of the full text. Upon detailed review, an additional 95 articles were excluded for reasons including coexisting pathology, conference abstracts, conservative treatment approaches, non-English language, irrelevant focus, and secondary complications. Thirty-seven studies met the inclusion criteria and were included in this systematic review.

### Inclusion and exclusion criteria

Table 2 shows the inclusion and exclusion criteria based on the study design, population and intervention. Studies that were published in English and described the surgical intervention and subsequent outcome of synovial chondromatosis of the knee were included. Conference abstracts and reviews were excluded in this study, but case reports and case series were included to capture a wider scope of the published literature.

**Table 2:** Inclusions and exclusion criteria.

Study characteristic	Inclusion criteria	Exclusion criteria
Study design	Case Reports, Case Series, Retrospective studies, Prospective studies	Non-English language publication Diagnostic studies Radiological reports/case reports
Population	Adult patients with Synovial Chondromatosis of the Knee	Pediatric patients Synovial Chondromatosis of other joints excluding the knee Concurrent pathologies coexisting in the knee joint
Interventions	Surgical intervention: synovectomy, arthroscopic procedures, other procedures	Conservative management Nonsurgical management No detailed surgical procedure
Outcome	Recurrence of Synovial Chondromatosis after operation	Recurrence of symptoms without looking for recurrence of disease

### Assessment of quality of included reviews

The ROBIN-I Tool was used to stratify risk of bias in nonrandomized studies of intervention as shown in Table 3.

**Table 3:** Assessment of quality of included reviews.

ROBINS-I tool for assessing risk of bias in nonrandomized studies								
Author, year of publication	Confounding risk	Selection risk	Intervention Classification risk	Deviation from intervention risk	Missing Data risk	Measurement of Outcome risk	Selection of reported result risk	Overall risk of bias
Coolican et al, 1989	Serious	Moderate	Low	Low	Moderate	Moderate	Low	Moderate
Ogilvie-Harris & Saleh, 1994	Moderate	Moderate	Low	Low	Low	Moderate	Low	Moderate
Ackerman et al, 2008	Moderate	Low	Low	Low	Low	Moderate	Low	Moderate
Houdek et al, 2017	Moderate	Moderate	Low	Low	Low	Low	Low	Moderate

### Data extraction

Data extraction was conducted in accordance with the Cochrane Handbook for Systematic Reviews of Interventions version 6.3.8 [23]. This includes summarizing the data in a prespecified format of: [1] author [2] year of publication [3] study characteristics including number of patients in each study, biodata including age and gender, the lesions reported on imaging studies and postoperative reports, the surgical technique(s) utilized, follow-up duration, and recurrence of synovial chondromatosis at follow-up. Statistical analysis was done using R statistical software [25]. Rate of recurrence was calculated as a percentage of cases recurred at the end of the follow-up period for each study.

The surgical techniques employed were be divided into open, arthroscopic, and combined approaches. Open approaches include arthrotomies without synovectomy, partial synovectomies, total synovectomies and TKA. Arthroscopic approaches involved arthroscopic washout alone, arthroscopic washout with partial synovectomy and arthroscopic washout with total synovectomy. Combined approaches involved 2-stage surgeries that included the use of both open synovectomy and arthroscopic washout.

Indications for surgical intervention were consistent across the included studies and were for symptomatic patient's refractory to conservative management. Arthroscopic procedures, including those with synovectomy and washout were generally chosen in cases where lesions were demonstrated to be intra-articular and well defined on imaging with accessibility through standard arthroscopic port sites. Open approaches were generally done in cases with extensive or florid intra-articular lesions, extra-articular lesions, or large lesions that would not be amenable to arthroscopic removal. Combined approaches were generally done in cases where arthroscopic removal was not possible in view of the size or location of the lesion, thus warranting an additional open approach to achieve adequate removal of lesions.

## Results

A total of 37 studies were included in this review comprising 30 case reports, 4 retrospective studies, and 3 case series. One hundred twenty patients were analyzed with 70 males and 49 females and 1 study which did not identify the gender of the patient. The mean age of intervention was 50.0 years old. Table 4 summarizes the included studies and the characteristics of each study.

**Table 4:** Summary of included studies.

Author, year of publication	Type of study	Number of patients (n)	Mean age (y)	Male (n)	Female(n)	Lesion
Abu-Yousef et al, 1985	Case Report	1	72	1	0	Well-encapsulated, measuring 4.5 × 4.5 × 3 cm in size in Popliteal Cyst
Aydin et al, 2012	Case Report	1	64	0	1	Bony structures as loose bodies (a main large 1 and 22 small ones) at the lateral compartment of the knee
Bozkurt et al, 2007	Case Report	1	58	0	1	Severe gonarthrosis of 4 compartments and loose bodies in the patellofemoral joint, lateral and medial tibiofemoral spaces and inferior recess of the proximal tibiofemoral joint
Butarbutar et al, 2024	Case Report	1	38	0	1	Cystic lesion (1.8 × 1.2 × 1.4 cm) was identified on the medial aspect of the right anteromedial condyle tibia. In addition, multiple intra-articular lesions were also observed in the tibiofemoral joint space, along with the effusion on the suprapatellar bursa
Church et al, 2006	Case Report	1	24	0	1	Extensive synovial fibrosis with a loose body, 4 cm × 4 cm, in the posterior compartment of the knee
Cirolia, 2017	Case Report	1	28	1	0	Multiple intra-articular calcified loose bodies with severe tricompartmental arthritis
Dogan et al, 2006	Case Report	1	51	1	0	multiple calcifications located posterior to the lateral femoral condyle, and posterior of Multiple 1-6 cm calcifications located posterior to the lateral femoral condyle and posterior of the tibial condyle extending to the gastrocnemius muscle
Dwidmuthe et al, 2017	Case Report	1	18	1	0	From the right knee joint, 5 large loose bodies ranging upto 4 cm in diameter with multiple small loose bodies removed. From the left knee joint, 3 large loose bodies ranging upto 2 cm in diameter with multiple small loose bodies removed
Fornaciari et al, 2015	Case Report	1	39	0	1	Heterogenous T2-weighted high signal intensity areas of calcification up to 5cm
Hill et al, 2024	Case Report	1	58	1	0	Intracapsular chondral nodules encountered with tricompartmental degenerative changes
Ho et al, 2019	Case Report	1	63	1	0	Intra-articular and Extra-articular loos bodies with stippled calcifications with a Baker's cyst.
Iyengar et al, 2007	Case Report	1	36	1	0	MRI revealed large effusion but no obvious synovial abnormality. Multiple cartilaginous loose bodies, and a large 3.3 cm piece of inflamed synovium with cartilaginous bodies, were seen arthroscopically
Jadawala et al. 2024	Case Report	1	24	-	-	MRI showed joint effusion, synovial hypertrophy and a loose calcific body just anterior of the distal femoral condyle causing pressure over the patellar tendon anteriorly as well as hyperdense cyst in the popliteal region. 2 × 3 cm loose body excised.
Kashyap et al, 2025	Case Report	1	35	1	0	Multiple intracapsular and extra-articular loose bodies; largest 3.5 cm × 2.5 cm
Khadkla et al, 2023	Case Report	1	47	1	0	MRI showed multiple loose bodies were seen intra- articularly and extending extra- articularly inside Baker's cyst
Kukreja, 2013	Case Report	1	16	1	0	MRI showing effusion, synovial hypertrophy and a loose calcific body in front of the femoral condyle pushing over the patellar tendon anteriorly. 7 × 4 cm loose body removed.
Lasmar et al, 2010	Case Report	1	34	1	0	Joint effusion with marked synovial proliferation. Suggestive of PVNS on MRI.
Limaiem et al, 2024	Case Report	1	68	1	0	18 cm × 15 cm × 6.5 cm calcific mass within the knee joint
Lin et al, 2016	Case Report	1	25	1	0	MRI with double meniscus sign suggestive of medial meniscal tear. Intraoperatively had loose bodies without any meniscal tear.
Osburn et al, 1990	Case Report	1	32	1	0	Discrete area of small punctate calcifications posterior to the knee thought not to involve the joint space
Osti et al, 2009	Case Report	1	52	1	0	CT showed presence of cartilaginous nodules into the Hoffa’s body area without bone erosion
Rai et al, 2022	Case Report	1	50	1	0	Multiple generalized calcified nodules intra-articularly and extra-articularly
Serbest et al, 2015	Case Report	1	72	1	0	MRI revealed many loose bodies (consistent with synovial chondromatosis) isointense with fatty bone marrow at all sequences at the level of right knee joint. Scalloping was observed at tibial and femoral surfaces. 20 × 1 × 6 cm lesion.
Shah et al, 2016	Case Report	1	43	1	0	MRI showed effusion, synovial hypertrophy and a loose calcific body behind the femoral condyle extending upto upper tibia
Shetty et al, 2025	Case Report	1	50	1	0	Ring and arc appearance on radiograph; 15 × 7 × 4 cm mass excised
Sourlas et al, 2013	Case Report	1	55	1	0	Radiographs showed 5 cm osteochondral lesion, located under the patella. Multiple loose bodies removed with the largest measuring 5.3 × 3 × 2.3 cm
Tomar et al, 2022	Case Report	1	40	0	1	MRI shows diffuse nodular synovial hypertrophy along the tibiofemoral joint, with multiple T2W hypointense foci showing blooming on GRE images within the knee joint
Tripathy et al, 2020	Case Report	1	24	1	0	Noncontrast CT showed aggregation of multiple calcific bodies within the suprapatellar bursa and within the knee joint suggestive of synovial chondromatosis.
Vasudevan et al, 2024	Case Report	1	24	0	1	MRI revealed multiple loose bodies in the patellofemoral and tibiofemoral joints
Zeleke et al, 2025	Case Report	1	50	1	0	Suprapatellar pouch and patellar tendon: 4 × 4 cm and 3 × 5 cm loose bodies. Posterior knee mass 5 x 4 cm.
Dorfmann et al, 1989	Case Series	29	43.2	18	11	10 Free loose bodies, 1 pedicled chondrome, 2 sessile forms, 9 free and pedicled, 10 free and sessile
Kyung et al, 2012	Case Series	2	52	1	1	Loose bodies around the posterior septum on MRI.
Primaputra et al, 2024	Case Series	4	58	0	4	Varied among patients
Ackerman et al, 2008	Retrospective Study	4	62	1	3	Not reported
Coolican et al, 1989	Retrospective Study	18	46	9	9	In 4 patients calcified cartilaginous lesions up to 1.5 cm in diameter were seen lying deep to the synovium. In 4 knees no abnormal synovium could be found but the joints were teeming with cartilage fragments. In 10 patients cartilage fragments from -.5 mm to 3 mm in diameter were seen either attached to synovial fringes or partially covered by synovium.
Houdek et al, 2017	Retrospective Study	20	63	12	8	Primary Synovial Chondromatosis
Ogilvie-Harris & Saleh, 1994	Retrospective Study	13	35	7	6	Varied among patients

CT, computed tomography; MRI, magnetic resonance imaging; GRE, gradient recalled echo.

Nineteen papers reported using an open approach to synovial chondromatosis. Of the 19 papers, there were 16 case reports, 1 case series, and 2 retrospective studies. A total of 44 knees were operated on with 25 male and 19 female patients and an average age of 48.1 years. A summary of the surgical technique, recurrence rate reporting, and mean follow-up duration are seen in Table 5.

**Table 5:** Open approaches surgical technique, recurrence reporting and follow-up duration.

Author, year of publication	Surgical technique	Recurrence reporting	Mean follow-up duration (y)
Fornaciari et al, 2015	Open Arthrotomy	No	20
Tripathy et al, 2020	Open Arthrotomy	No	1
Aydin et al, 2012	Open Lateral Partial Meniscectomy	No	2
Bozkurt et al, 2007	Open Partial Synovectomy via medial parapatellar approach	No	1
Rai et al, 2022	Open Radical Synovectomy	No	4
Dwidmuthe et al, 2017	Open Synovectomy	No	0.5
Limaiem et al, 2024	Open Synovectomy	No	0.33
Osburn et al, 1990	Open Synovectomy	Yes 2 mo later found recurrent calcifications and treated with physiotherapy. 4 y later had extensive recurrence.	4
Serbest et al, 2015	Open Synovectomy	No	Not specified
Shah et al, 2016	Open Synovectomy	No	1
Shetty et al, 2025	Open Synovectomy	No	2
Kukreja, 2013	Open Synovectomy via medial parapatellar approach	No	1
Dogan et al, 2006	Open Synovectomy via posterior approach	No	0.75
Abu-Yousef et al, 1985	Open Total Synovectomy	No	2
Church et al, 2006	Open Total Synovectomy	Yes: Radical open synovectomy done 2 y later. Subsequent arthroscopic debridement. At end of follow-up diagnosed with recurrent synovial chondromatosis	5
Ackerman et al, 2008	TKA	1: Subsequent Synovectomy	4.9
Houdek et al, 2017	TKA	0 out of the 4 who never had any previous operation. 5 out of the 15 with previous operations besides a TKA	25
Primaputra et al, 2024	TKA (2 patients); Open Synovectomy (2 patients)	No	0.37
Zeleke et al, 2025	Two-stage Open Radical Synovectomy—first stage medial parapatellar approach; second stage posterior approach	No	1

When choosing an open approach, studies either performed [1] Open Arthrotomy [26,27] with removal of loose bodies without synovectomy [2] Open Synovectomy (Partial or Total) [11,[28], [29], [30], [31], [32], [33], [34], [35], [36], [37], [38], [39], [40]] or [3] TKA [15,38,41]. Of the 3 open approaches, the rate of recurrence is listed in Table 6.

**Table 6:** Open approaches recurrence rate.

Type of surgery	Number of knees	Number of recurrences	Rate of recurrence
Open Arthrotomy	2	0	0%
Open Synovectomy	16	2	12.5%
TKA	26	6	23.1%

Open arthrotomy without synovectomy did not have any recurrence (follow-up duration 1 and 20 years), open synovectomy had a 12.5% recurrence rate (2 out of 16 cases; average follow-up duration 2.04 years) and TKA had a recurrence rate of 20.8% (6 out of 26 cases; average follow-up duration 10.3 years). For the open arthrotomy group, given the small sample size (2 knees) and the lack of synovectomy, these cases may not reliably estimate treatment effect.

Zeleke et al (2025) [42] took a unique 2-stage approach in removing loose bodies in the suprapatellar pouch and the posterior knee separately 6 weeks apart and found no recurrence after 1 year.

Primaputra et al (2024) [38] performed a comparison study in a case series of 4 patients with 2 patients undergoing TKA and 2 patients undergoing Open Synovectomy and found that postoperative Musculoskeletal Tumor Society score scores were slightly higher (indicating better outcomes) for synovectomy alone compared to synovectomy with total knee replacement.

### Arthroscopic approaches

Twelve papers reported using an arthroscopic approach to the management of synovial chondromatosis. Of the 12 papers, there were 8 case reports, 2 case series, and 2 retrospective studies. A total of 70 knees were operated on with 40 male and 29 female and 1 study which did not report the gender of the patient. The average age was 38.6 years old. A summary of the surgical technique, recurrence rate reporting, and mean follow-up duration are seen in Table 7.

**Table 7:** Arthroscopic approaches surgical technique, recurrence reporting, and follow-up duration.

Author, year of publication	Surgical technique	Recurrence reporting	Follow-up duration (y)
Cirolia, 2017	Arthroscopic Total Synovectomy and loose body excision	No recurrence	0.25
Coolican et al, 1989	Arthroscopic Synovectomy	1 out of 18 recurred.	3.5
Dorfmann et al, 1989	Arthroscopic removal of loose bodies without synovectomy	7 out of 29 patients	3.5
Hill et al. 2024	Arthroscopic evacuation with radical synovectomy	No recurrence	1 y
Iyengar et al, 2007	Arthroscopic removal of loose bodies with synovectomy	No recurrence	0.75
Jadawala et al. 2024	Arthroscopic debridement with synovectomy	No recurrence	0.42
Kyung et al, 2012	Arthroscopic synovectomy and partial meniscectomy and removal of loose bodies	No recurrence (0 out of 2 patients)	1.25
Lin et al, 2006	Arthroscopic washout with partial synovectomy	No recurrence	1.66
Ogilvie-Harris & Saleh, 1994	Arthroscopic washout vs Arthroscopic synovectomy with removal of loose bodies	3 out of 5 of washout only patients recurred. None of the 8 synovectomy patients recurred.	3.17
Osti et al, 2009	Arthroscopic removal by partial excision of Hoffa's Body	Yes, 3 y later underwent subsequent medial mini-arthrotomy and arthroscopic washout	13
Tomar et al, 2022	Arthroscopic Subtotal Synovectomy and Debridement	Yes, 3 mo later and an open synovial debridement and redo biopsy were advised to also rule out malignant transformation. Patient did not want further intervention and was lost to follow-up	0.5
Vasudevan et al, 2024	Arthroscopic extraction of loose bodies with synovectomy	No recurrence	0.25

When performing an arthroscopic approach, studies either performed [1] Arthroscopic removal of loose bodies without synovectomy/ Arthroscopic Washout [2,43,44] Arthroscopic removal of loose bodies with synovectomy (partial vs total) [[45], [46], [47], [48], [49], [50], [51], [52], [53]]. The rate of recurrence of arthroscopic approaches are listed in Table 8.

**Table 8:** Arthroscopic approaches recurrence rate.

Type of surgery	Number of knees	Number of recurrences	Rate of recurrence
Arthroscopic removal of loose bodies/ washout without synovectomy	35	11	31.4%
Arthroscopic removal of loose bodies with synovectomy	35	2	5.71%

Arthroscopic removal of loose bodies had a recurrence rate of 31.4% (11 out of 35 cases; average follow-up duration 6.56 years) while arthroscopic removal of loose bodies with synovectomy had a lower recurrence rate of 5.71% (2 of 35 cases; average follow-up duration 1.53 years).

Ogilvie-Harris & Saleh (1994) [19] performed a comparison between an arthroscopic washout and arthroscopic removal of loose bodies with synovectomy and found that patients that underwent only a washout had recurrence of synovial chondromatosis (3 out of 5 patients) while those with total synovectomy (8 patients) did not experience any recurrence.

### Combined approaches

Six case reports reported the use of combined arthroscopic work and an open approach in the management of synovial chondromatosis.

Ho et al (2019) [54] and Khadka et al (2023) [55] reported the use of a 2-stage operation for synovial chondromatosis intra-articularly and in Baker's Cyst. The first stage involved open excision of Baker's cyst and subsequently an arthroscopic removal of intra-articular loose bodies was performed. Ho et al (2019) [54] and Khadka et al (2023) [55] did not report any recurrence of synovial chondromatosis at 3 years of follow-up and 1 year of follow-up respectively.

Butarbutar et al (2024) [56] also opted for a 2-stage approach with the first stage involving an arthroscopic synovectomy and the second stage involving removal of a cartilage-like mass measuring 3.5 × 3 cm through an open incision. They did not report any recurrence at 1 year of follow-up.

Lasmar et al (2010) [57] performed a single stage arthroscopy for removal of loose bodies and an open arthrotomy with partial synovectomy. No recurrence was reported at 8 months of follow-up.

Sourlas et al (2013) [58] performed a single-stage arthroscopic washout to remove loose bodies and proceeded intraoperatively with a medial parapatellar arthrotomy and total synovectomy given that there was a giant loose body that could not be removed arthroscopically. There was no recurrence reported at 2.5 years of follow-up.

Kashyap et al (2025) [59] performed a diagnostic arthroscopy revealing osteochondromas and loose bodies globally within the joint space. Partial synovectomy was performed with loose body removal and a subsequent limited sura-patellar arthrotomy was done using a direct anterior midline approach for larger extra-articular loose bodies. There was no recurrence reported at 6 months of follow-up.

There was no recurrence among the 6 patients reported across the 6 case reports of a combined approach with an average follow-up duration of 1.44 years.

## Discussion

This study has compiled and studied the recurrence rates associated with different surgical techniques. Open approaches, including open arthrotomy, open synovectomy, and TKA, had varying recurrence rates. Open arthrotomy demonstrated no recurrence; however, given the small sample size of 2 cases, it might not be generalizable given the existing literature advocating for synovectomy to reduce the risk of recurrence [30]. However, open synovectomy showed a 12.5% recurrence rate, and TKA had a recurrence rate of 20.8%. Singh et al [60] posit that an open synovectomy has been a preferred option in extensive synovial chondromatosis of the knee, as an open procedure gives better chances for disease eradication than the arthroscopic one. Our review aligns with other reports indicating recurrence rates for TKA for primary synovial chondromatosis being that of 3%-25% [[61], [62], [63]]. McKenzie et al. [63] have suggested that TKA recurrence would be due to incomplete surgical resection.

Arthroscopic approaches, which include arthroscopic washout and synovectomy, showed a lower overall recurrence rate. The higher recurrence rate observed with arthroscopic washout alone (31.4%) compared to arthroscopic synovectomy (5.71%) may be hypothesized to be due to incomplete removal of loose bodies. This suggests that, regardless of the approach, meticulous excision of both loose bodies and diseased synovium is critical in minimizing recurrence. Arthroscopic approaches have been gaining traction as they have minimal surgical trauma and impact on joint function postoperatively relative to open procedures [64].

The combined approaches, though reported in fewer cases, showed no recurrence. This might be attributed to the comprehensive nature of these procedures, combining the benefits of both open and arthroscopic methods to ensure thorough removal of loose bodies and affected synovium. The use of both arthroscopic and open approaches allows the surgeon to visualize the extent of disease and decide [64] whether to proceed with an arthroscopic wash out and synovectomy or to convert to an open procedure through a mini-arthrotomy to avoid incomplete excision through arthroscopic approaches [58]. However, given the short follow-up duration of the cases reported, under-reporting of recurrence may be a factor to consider.

Based on the existing literature, arthroscopic treatment for synovial chondromatosis is preferred for intra-articular manifestations of disease while open approaches reserved for extra-articular or extensive involvement [19,54,65,66] Our study found that a combined approach may be beneficial in aiding the surgeon make an informed decision given the low recurrence rate of using a combination of arthroscopy and open techniques and the flexibility of allowing for decision making intraoperatively. However, given the variability of the lesions, many included studies had justified their rationale for either technique based off imaging modalities, surgeon preference, and clinical acumen.

Several limitations should be considered when interpreting our findings. First, the included studies were predominantly case reports and retrospective studies, which inherently carry a risk of bias and limited generalizability. The sample sizes were small, and the follow-up periods varied, which may affect the reported recurrence rates. Additionally, the heterogeneity in surgical techniques, patient populations, and outcome measures across studies posed challenges in synthesizing the data. However, this study attempts to summarize the various lesions presented in the literature and the relevant approaches that the surgeons ultimately undertook, synthesizing the recurrence and variety of operative options available for surgeons. Lastly, the varying follow-up periods of each study may lead to under-reporting of recurrence in studies which had shorter follow-up periods.

## Conclusions

This systematic review underscores the importance of surgical intervention in the management of synovial chondromatosis of the knee. While open arthrotomy and arthroscopic synovectomy demonstrate lower recurrence rates, TKA and arthroscopic washout alone are associated with higher recurrence. The findings suggest that a comprehensive approach individualized to patient-specific lesions should be considered and based on imaging modalities and surgeon preference, purely intra-articular lesions should be managed arthroscopically but, in the situation where extra-articular manifestation is present, combining open and arthroscopic techniques might be the most effective in preventing recurrence.

## Conflicts of interest

The authors declare there are no conflicts of interest.

For full disclosure statements refer to https://doi.org/10.1016/j.artd.2025.101796.

## CRediT authorship contribution statement

**Zhen Jonathan Liang:** Writing – review & editing, Writing – original draft, Validation, Project administration, Methodology, Investigation, Formal analysis, Data curation, Conceptualization. **Yu Liu:** Writing – review & editing, Writing – original draft, Validation, Supervision, Investigation, Formal analysis, Conceptualization. **Vachalam Rangasamie Danakkrisna:** Writing – review & editing, Validation, Supervision, Conceptualization. **Zi Qiang Glen Liau:** Writing – review & editing, Writing – original draft, Supervision, Methodology, Investigation, Formal analysis, Data curation, Conceptualization.

## Appendix A Supplementary data

Conflict of Interest Statement for Vachalam Danakkrisna

Conflict of Interest Statement for Liau Zi

Conflict of Interest Statement for Liu

Conflict of Interest Statement for Liang
