# Supplementary material for: Surgical Options for Primary Synovial Chondromatosis of the Knee: A Systematic Review
Source: Arthroplast Today. 2025 Aug 13;35:101796. doi: 10.1016/j.artd.2025.101796 (PMC12361623; doi:10.1016/j.artd.2025.101796)
Supplement: Conflict of Interest Statement for Liu [file mmc3.docx]

# CONFLICT OF INTEREST STATEMENT

***American Association of Hip and Knee Surgeons***

(Adopted from the American Academy of Orthopaedic Surgeons disclosure statement)

The following form **must be filled out completely and submitted by each author (example, 6 authors, 6 forms).**

**All items require a response. If there is no relevant disclosure for a given item, enter "*None*.”**

Manuscript Title: Surgical Options for Primary Synovial Chondromatosis of the Knee – A Systematic Review

1. Royalties from a company or supplier (The following conflicts were disclosed)

No royalties were received.

2. Speakers bureau/paid presentations for a company or supplier (The following conflicts were disclosed)

No payment was made.

3A. Paid employee for a company or supplier (The following conflicts were disclosed)

Not a paid employee.

3B. Paid consultant for a company or supplier (The following conflicts were disclosed)

Not a consultant for company or supplier.

3C. Unpaid consultants for a company or supplier (The following conflicts were disclosed)

Not a consultant for company or supplier.

4. Stock or stock options in a company or supplier (The following conflicts were disclosed)

Not holding any stocks or options from the company or supplier.

5. Research support from a company or supplier as a Principal Investigator (The following conflicts were disclosed)

Not a research support from the company or supplier.

6. Other financial or material support from a company or supplier (The following conflicts were disclosed)

No financial or material support from company or supplier.

7. Royalties, financial or material support from publishers (The following conflicts were disclosed)

No royalties received.

8. Medical/Orthopaedic publications editorial/governing board (The following conflicts were disclosed)

Not from any publications editorial or governing board.

9. Board member/committee appointments for a society (The following conflicts were disclosed)

Not a board member or holding any appointments for any society.

**Each author must sign AND print or type his/her name, date and submit a separate form**

In addition, one BLINDED Conflict of Interest form (no author names used) should be submitted per manuscript with all author disclosures.

18/4/2025


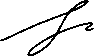


YU LIU

Author Name (Print or Type) Author Signature Date
